# Seasonal Cholera from Multiple Small Outbreaks, Rural Bangladesh

**DOI:** 10.3201/eid1405.071116

**Published:** 2008-05

**Authors:** O. Colin Stine, Munirul Alam, Li Tang, G. Balakrish Nair, A. Kasem Siddique, Shah M. Faruque, Anwar Huq, Rita Colwell, R. Bradley Sack, J. Glenn Morris

**Affiliations:** * University of Maryland School of Medicine, Baltimore, Maryland, USA; † International Center for Diarrheal Disease Research, Dhaka, Bangladesh; ‡ University of Maryland, College Park, Maryland, USA; § Johns Hopkins Bloomberg School of Public Health, Baltimore, Maryland, USA; ¶ University of Florida, Gainesville, Florida, USA

**Keywords:** Cholera, variable number of tandem repeats, epidemiology, outbreaks, genetic variation, dispatch

## Abstract

Clinical and environmental *Vibrio cholerae* organisms collected from February 2004 through April 2005 were systematically isolated from 2 rural Bangladeshi locales. Their genetic relatedness was evaluated at 5 loci that contained a variable number of tandem repeats (VNTR). The observed minimal overlap in VNTR patterns between the 2 communities was consistent with sequential, small outbreaks from local sources.

Cholera is a major cause of illness in the developing world. The World Health Organization reported in 2006 that 236,896 cases of cholera occurred in 52 countries, a 79% increase over 2005 (*1*). Although major advances in the understanding of the molecular basis of *Vibrio cholerae* pathogenicity have been made, including defining the environmental reservoirs for the microorganism (*2*–*4*), we do not fully understand the cause of seasonal epidemics in cholera-endemic areas nor the factors that drive epidemics. Specifically, whether these seasonal epidemics arise from a single clonal strain or reflect superimposition of multiple small outbreaks is not clear.

## The Study

From February 2004 through April 2005, we systematically collected clinical and environmental *V. cholerae* from Bakerganj and Mathbaria, 2 small communities 50 miles apart in the southern part of coastal Bangladesh. Samples were collected on 3 consecutive days every 2 weeks throughout the year. Clinical isolates were collected from ≈20% of all patients who had symptoms of cholera when seen at the local clinics. Environmental isolates were cultured from water, sediment, and plankton samples taken at 6 sites (ponds or river sites) in each of the 2 communities. The same sites were used throughout the 15-month study, and the same method was applied at all sites and across all time points. Isolation was performed by standard culture methods, and *V. cholerae* was identified by a combination of biochemical (*5*), molecular, and serologic techniques (*6*). All samples were collected according to protocols approved by Institutional Review Boards at Johns Hopkins University, University of Maryland, and the International Centre for Diarrheal Disease Research, Bangladesh.

For multilocus sequence typing (MLST) and variable number of tandem repeat (VNTR) determinations, each locus was PCR amplified by using standard conditions and appropriate primers from the literature (*7*) (Technical Appendix). The resulting fragments were sequenced by using Big Dye Kit (Applied Biosystems, Foster City, CA, USA). Trace files were generated by using an ABI 3730xl automatic sequencer and read using either 1) the Phred (*8*,*9*), Phrap (www.washington.edu), or Consed (*10*) package or 2) Sequencher (AGCT, Gene Codes Corporation, Ann Arbor, MI, USA).

A total of 391 environmental and clinical isolates of *V. cholerae* were collected and identified from February 2004 through April 2005. Of these, 267 environmental isolates were identified as belonging to non-O1 and non-O139 serogroups and did not carry the gene for cholera toxin (*ctx*). Analysis of these 267 by MLST (using the 7 loci identified previously [*7*]) yielded a genetic background that was distinct from that of the clinical/epidemic strains. The other 68 (20%) of 335 environmental *V. cholerae* isolates shared a genetic background identical or nearly identical to clinical/epidemic *V. cholerae.* These 68 and all 56 clinical isolates collected (all of which were related by MLST) were further analyzed by examining 5 VNTR loci.

Sequence typing was based on 5 polymorphic VNTR loci. These loci were identified with the program Tandem Repeat Finder (*11*). Four of the 5 loci had hexameric repeats in coding regions. The loci were identified by those genes in which they occur: VC0147vntr, VC0436–7vntr (intergenic), VC1650vntr, VC0171vntr, and VCA0283vntr. Alleles were distinguished by the number of tandem repeats as determined by Tandem Repeat Finder (*11*) (Technical Appendix). Sequences from 1 locus with identical numbers of repeats were assigned to the identical allele. The alleles at the 5 loci were ordered to generate a sequence type (ST), for example, 3,5,2,2,8. Each locus was polymorphic with 7, 6, 6, 20, and 16 alleles, respectively. Thirty-six STs were observed. The various STs were defined as related if they were identical at 4 of the 5 loci. When we defined a VNTR genetic group as differing by a single locus variant from another member of the group, 3 large VNTR genetic groups were identified and 5 VNTR genetic groups composed of only 2 isolates and 7 unrelated strains. These 7 singletons differed from all other STs at 2 or more loci.

There was statistically significant agreement between serogroup and VNTR genetic group. For *V. cholerae* O139, all STs were 4,1,1,x,x (Technical Appendix). Thus, the isolates were considered to be related because x,x = 1,1; 2,1; or 2,8, i.e., a change in a single locus serially connected all isolates. Summing the number of isolates of a sequence type, we found that the 23 ctx^+^ O139 strains formed a VNTR genetic group. A second group comprised 75 ctx^+^ O1 Inaba isolates. Finally, 18 ctx^+^ O1 Ogawa clustered into 3 additional VNTR genetic groups. There were 10 exceptions, i.e., 3 non-O1, non-O139 ctx^+^ isolates were in groups; 3 ctx^–^ O139, 2 ctx^–^ O1 Inaba, 1 ctx^–^, and 1 ctx^+^ O1 Ogawa were not.

We found that Bakerganj and Mathbaria yielded distinct *V. cholerae* populations; only 2 (ST 3,5,2,2,7 and 1,1,3,9,8) of 36 STs identified were found at both locations (Table 1; Technical Appendix). There was substantial divergence in STs among strains isolated from patients, compared with strains from the environment in Mathbaria; only 1 (ST 3,5,2,2,7) of 16 STs were found in both patient and environmental isolates. Similarly, in Bakerganj, only 2 (ST 3,5,2,2,6 and 3,5,2,1,5) of 24 STs were found in both clinical and environmental isolates.

**Table 1:** Number of *Vibrio cholerae* sequence types in distinct serotypes and sample types in Bakerganj and Mathbaria, Bangladesh, 2004–2005

Serotype	Source	Bakerganj	Mathbaria
O1 Inaba	Clinic	10	7
	Environment	7	1
O1 Ogawa	Clinic	6	6
	Environment	1	0
O139	Environment	0	3

Clinical or environmental isolates from a given period were more likely to have a common ST (Technical Appendix). For example, at Mathbaria, 49 of the 53 isolates with an ST identical to that of another isolate were found in the same or neighboring month. Similarly, at Bakerganj, 33 of 36 isolates with identical STs were found in the same or neighboring month.

Variation in the VNTR loci appeared to be greater among clinical isolates than among environmental isolates. A total of 29 STs occurred in clinical isolates, whereas only 12 occurred in environmental isolates (Table 1). When we controlled for location and month of collection (Table 2), the total number of STs among environmental isolates (7 ST/35 isolates) was less than that among clinical isolates (16 ST/32 isolates) (χ^2^ = 4.4, df 1, p = 0.036). Common STs were found among environmental isolates, despite the isolates coming from samples from different ponds and distinct subsamples (e.g., water, phytoplankton, zooplankton).

**Table 2:** Sequence type (ST) variations among *Vibrio cholerae* O1 Inaba isolates from environmental and clinical sources by month of collection, Bangladesh, 2004–2005

Location	Date	Source	No. ponds	No. isolates	No. STs	Variation*
Mathbaria	2004 Dec	Environment	4	12	1	0.08
Bakerganj	2004 Sep	Environment	5	16	4	0.25
Bakerganj	2005 Apr	Environment	4	7	2	0.29
Bakerganj	2004 Oct	Clinic		9	4	0.44
Mathbaria	2004 May	Clinic		11	5	0.45
Mathbaria	2004 Apr	Clinic		8	4	0.50
Bakerganj	2004 Dec	Clinic		4	3	0.75

*Variation, no. STs/no. isolates.

## Conclusions

Our data do not support the concept of seasonal cholera epidemics occurring by movement of a single clonal wave across the countryside. They are consistent, instead, with the natural occurrence of *V. cholerae* year-round in the aquatic environment of each site, with each site having its own, distinct grouping of strains (*12*,*13*). The limited overlap between STs in environmental and clinical isolates is an enigma that remains to be resolved. However, the extensive variation in VNTR STs in this short time frame and small geographic area suggests that VNTR STs can be useful in assessing genetic relatedness of isolates during outbreaks/epidemics. The strong temporal clustering of the variation arising in the VNTR STs of clinical isolates is consistent with the hypothesis that clinical cases reflect the occurrence of multiple small outbreaks.

Our data are drawn from rural Bangladesh; however, cholera is a global disease. Its epidemiology may well differ in sub-Saharan Africa, the Americas, or other parts of Asia, or in the mega-cities that are increasingly the hallmark of the developing world. These variations emphasize the need for application of similar techniques in these diverse settings.

## Supplementary Material

Technical Appendix
