# Supplementary material for: Seasonal Cholera from Multiple Small Outbreaks, Rural Bangladesh
Source: Emerg Infect Dis. 2008 May;14(5):831–3. doi: 10.3201/eid1405.071116 (PMC2600222; doi:10.3201/eid1405.071116)

Technical Appendix

Primers for 5 variable number of tandem repeat loci in *Vibrio cholera*:

Gene	Basepair	Primer (5′ → 3′)
VC0147	136981	TTGTCATGGCTTGGATTTGG
	137461	TGTCGATCACCAATGGCTGC
VC0436-7	466861	CGTGGTACTAAGTTCCACGC
	467401	CGTTTTTACCACGCTCCGCTTC
VC1650	1778221	CTACCAAGCGGCGGTTAAGCTG
	1778701	TGGGCAACCTGCTGGTAGC
VCA0171	187561	GCATCATCCACAGCGTTTGG
	188101	GCTGAAGCCTTTCGCGATCC
VCA0283	303781	GTACATTCACAATTTGCTCACCC
	304201	ACTTCAAAACTATTGCGCAC

Standard conditions were a 30-μL reaction containing 100 μmol/L of each dNTP, 1.5 mmol/L $MgCl_2$, 1× PCR buffer (Invitrogen, Carlsbad, CA, USA), and 1 unit of Taq DNA polymerase (Invitrogen); cycling was 30 times at 95°C for 30 s, annealing (at 50°C or 55ºC) for 30 s and 72°C for 30 s.

ClustalX (*1*) alignment of 6 sequences derived from *Vibrio cholerae* isolates to determine distinct alleles. Each row represents a single isolate. Each base is a different color. The repeating unit is the hexamer: AACAGC. The dashes mark bases missing relative to the longest repeat in the first row.

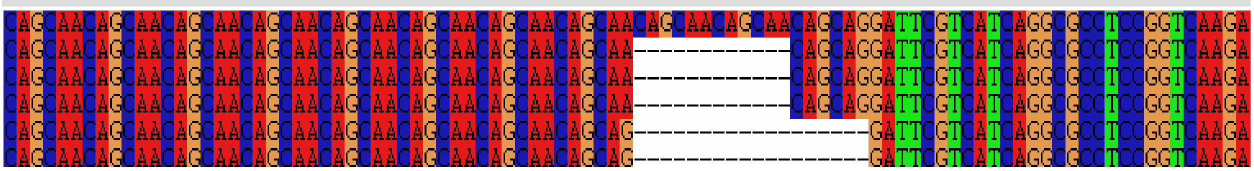

Row 1 = allele 1; rows 2, 3, and 4 = allele 2; rows 5 and 6 = allele 3

Distribution of sequence types by serotype and source in Bakerganj and Mathbaria. For each sequence type, the source is indicated by a C for clinical or an E for environmental, the number of isolates follows after n=, and the month(s) when it was isolated. The arrows indicate sequence types found at both locales, while the barbells indicate sequence types found in both clinical and environmental samples within a locale.

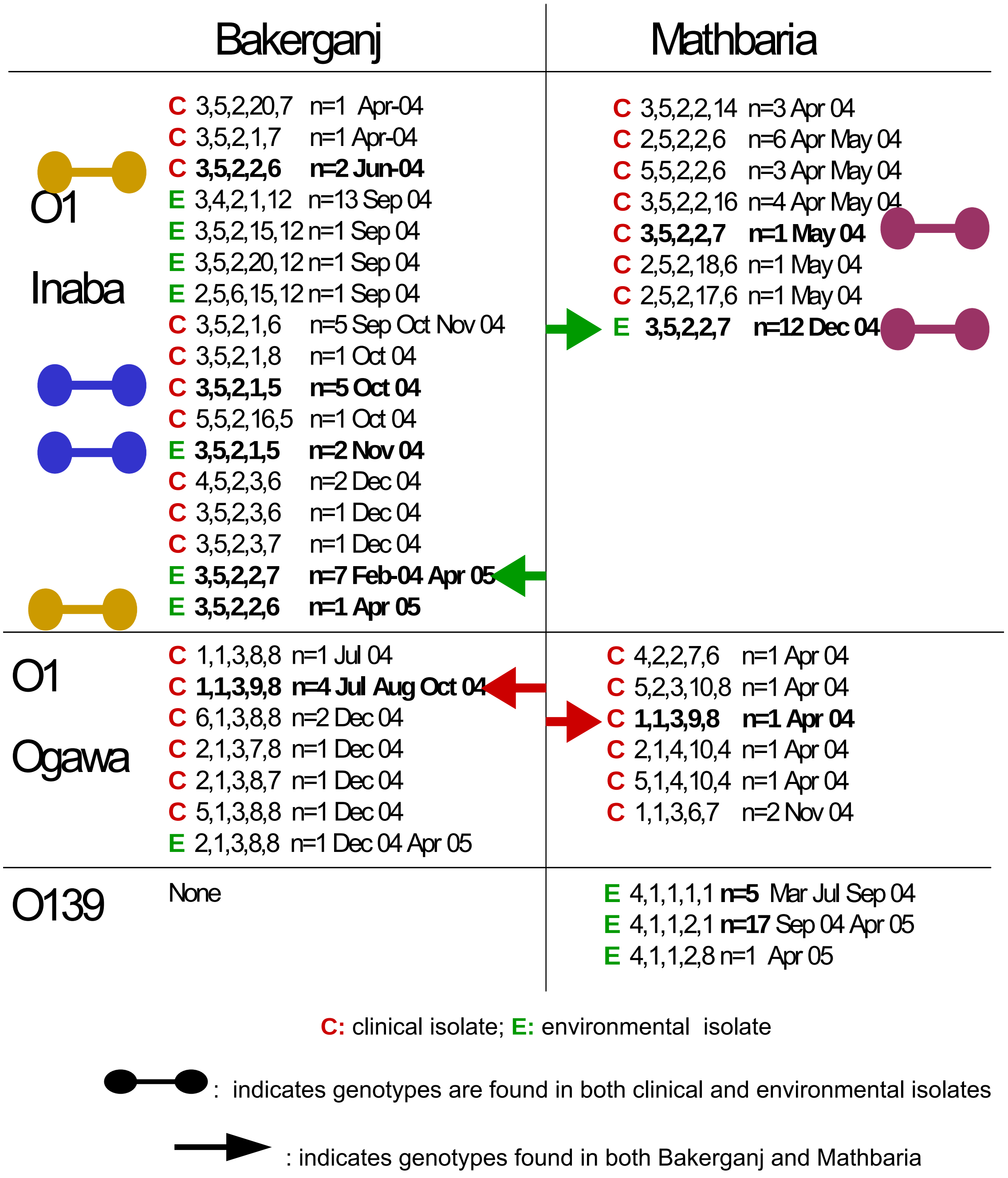

Supplement: Technical Appendix [file 07-1116_Techapp-s1.pdf]
